# Endobronchial ultrasound is feasible and safe to diagnose pulmonary embolism in non-transportable SARS-CoV-2 ARDS patients requiring extracorporeal lung support

**DOI:** 10.1186/s13054-020-03292-9

**Published:** 2020-09-21

**Authors:** Maxens Decavele, Valery Trosini-Désert, Samia Boussouar, Baptiste Duceau, Martin Dres, Alexandre Demoule

**Affiliations:** 1 AP-HP, Groupe Hospitalier Universitaire APHP-Sorbonne Université, site Pitié-Salpêtrière, Service de Pneumologie, Médecine Intensive et Réanimation (Département R3S), F-75013 Paris, France; 2 Sorbonne Université, INSERM, UMRS1158 Neurophysiologie Respiratoire Expérimentale et Clinique, F-75005 Paris, France; 3 AP-HP, Unité d’imagerie cardio-thoracique, hôpitaux universitaires Pitié-Salpêtrière Charles Foix, AP-HP, 75013 Paris, France; 4 Département d’anesthésie réanimation, AP-HP, Sorbonne Université, Hôpital Pitié-Salpêtrière, Paris, France

Dear editor,

The SARS-CoV-2 (severe acute respiratory syndrome coronavirus 2)-related acute respiratory distress syndrome (ARDS) is associated with an elevated coagulation activation pattern [1] and a high incidence of pulmonary embolism [2]. The diagnosis of pulmonary embolism (PE) may be challenging in these patients because computed tomography pulmonary angiogram (CTPA) requires an intrahospital transport with potential adverse effects and also may increase the risk of acute kidney failure (contrast-induced nephropathy). This is even more the case in up to 10% of SARS-Cov-2 ARDS patients who require venovenous extracorporeal membrane oxygenation (vv-ECMO) as an extracorporeal lung support [1]. In addition to the inherent risks of hospital transport, which are particularly high in these patients [3], extracorporeal circulation is likely to alter the quality of the contrast agent distribution and may reduce the diagnostic performance of the CTPA [4]. Finally, systematic curative antithrombotic therapy is not a safe option as it exposes to a serious risk of bleeding, especially when prolonged vv-ECMO is expected [5]. For all the abovementioned reasons, alternative techniques allowing the diagnosis of PE in these vv-ECMO patients would be of the highest interest.

Here, we describe the feasibility, safety, and diagnostic accuracy of endobronchial ultrasound (EBUS) to detect PE in patients with severe SARS-CoV-2 ARDS requiring vv-ECMO. Between April 15 and May 1, 2020, eleven patients were included. The procedure was performed using a 6.7-mm-outer-diameter, real-time, bronchoscope (EB-530US; FUJIFILM Medical Corporation, Tokyo, Japan) with a 7.5-MHz linear ultrasound transducer (SU-1 H; FUJIFILM Medical Corporation, Tokyo, Japan) equipped with color-Doppler. For each patient, EBUS procedure followed the same roadmap [6]. All EBUS images and videos were reviewed by two independent experts in thoracic radiology (S.B. and D.T.) blind from the CTPA interpretation. The study was approved by the research ethics committee of Sorbonne University (CER-SU N°2020-48) and information was given to the patients or their relatives.

Patients were mostly men (*n* = 10), 52 [49–55] years old, with a body mass index of 29 [28–31] kg/m^2^ (Table 1). The time between intubation, vv-ECMO and EBUS was 21 [11–27] and 13 [7–18] days, respectively. At the time of EBUS procedure, three patients were not receiving antithrombotic therapy, two were receiving effective curative unfractionated heparin, and six were receiving prophylactic unfractionated heparin (dose was 18,000 [14,000–20,000] UI/day). Pulmonary embolism was observed on EBUS in five of the eleven patients (45%) (Fig. 1). The duration of the procedure was 15 [13–17] min and no major adverse effect of EBUS (e.g., serious bleeding, arterial oxygen saturation < 85%) was reported. EBUS could explore part of segmental arteries in five (45%) patients.

**Table 1:** Patients’ characteristics and diagnostic correspondence between Endobronchial Ultrasound (EBUS) and Computed tomography pulmonary angiography (CTPA)

Patient age (years old)	Ventilator settings, PEEP (cmH_2_O) FiO_2_	EBUS duration (min)	Lowest SpO_**2**_ during EBUS	V**t** before EBUS (mL)	V**t** during EBUS (mL)	CTPA performed before or after EBUS	Time between EBUS-CTPA (days)	Location of PE on EBUS	Location of PE on CTPA	Agreement EBUS-CTPA
**Patient 1** 54	**PEEP:** 14 **FiO**_**2**_**:** 100%	17	90	80	60	After	17	SBRPA	SBRPA	**Yes**
**Patient 2** 55	**PEEP:** 12 **FiO**_**2**_**:** 80%	15	94	72	45	Before	8	–	Ap-SBLPA Distal P-SBRPA	No
**Patient 3** 61	**PEEP**: 8 **FiO**_**2**_**:** 70%	17	94	480	480	Before	6	SBLPA	SBLPA Segmental IBRPA	**Yes**
**Patient 4** 46	**PEEP:** 12 **FiO**_**2**_**:** 30%	21	95	71	35	Before	7	A-SBRPA	–	No
**Patient 5** 51	**PEEP:** 12 **FiO**_**2**_**:** 30%	13	98	90	37	Before	10	–	–	**Yes**
**Patient 6** 57	**PEEP:** 12 **FiO**_**2**_**:** 70%	11	96	30	25	After	5	Ap-SBRPA	Ap-SBRPA	**Yes**
**Patient 7** 35	**PEEP:** 12 **FiO**_**2**_**:** 50%	14	94	430	250	Before	6	–	–	**Yes**
**Patient 8** 35	**PEEP:** 12 **FiO**_**2**_**:** 50%	15	100	67	32	Before	7		**–**	**Yes**
**Patient 9** 68	**PEEP**: 10 **FiO**_**2**_**:** 50%	18	100	380	350	Before After	7 5	– IBRPA	– IBRPA	**Yes** **Yes**
**Patient 10** 68	**PEEP:** 12 **FiO**_**2**_**: 50%**	13	92	220	150	After	9		–	**Yes**
**Patient 11** 39	**PEEP:** 12 **FiO**_**2**_**:** 60%	11	88	50	35	After	1		–	**Yes**

*PEEP* positive end-expiratory pressure, *Vt* tidal volume, *PE* pulmonary embolism, *SBRAP* superior bronchial right pulmonary artery, *A-SBRPA* anterior segment of the SBRPA, *P-SBRPA* posterior segment of the SBRPA, *Ap-SBRPA* apical segment of the SBRPA, *LPA* left pulmonary artery, *IBLPA* inferior bronchial left pulmonary artery, *SBLPA* superior bronchial left pulmonary artery, *T* trachea

**Fig. 1:**
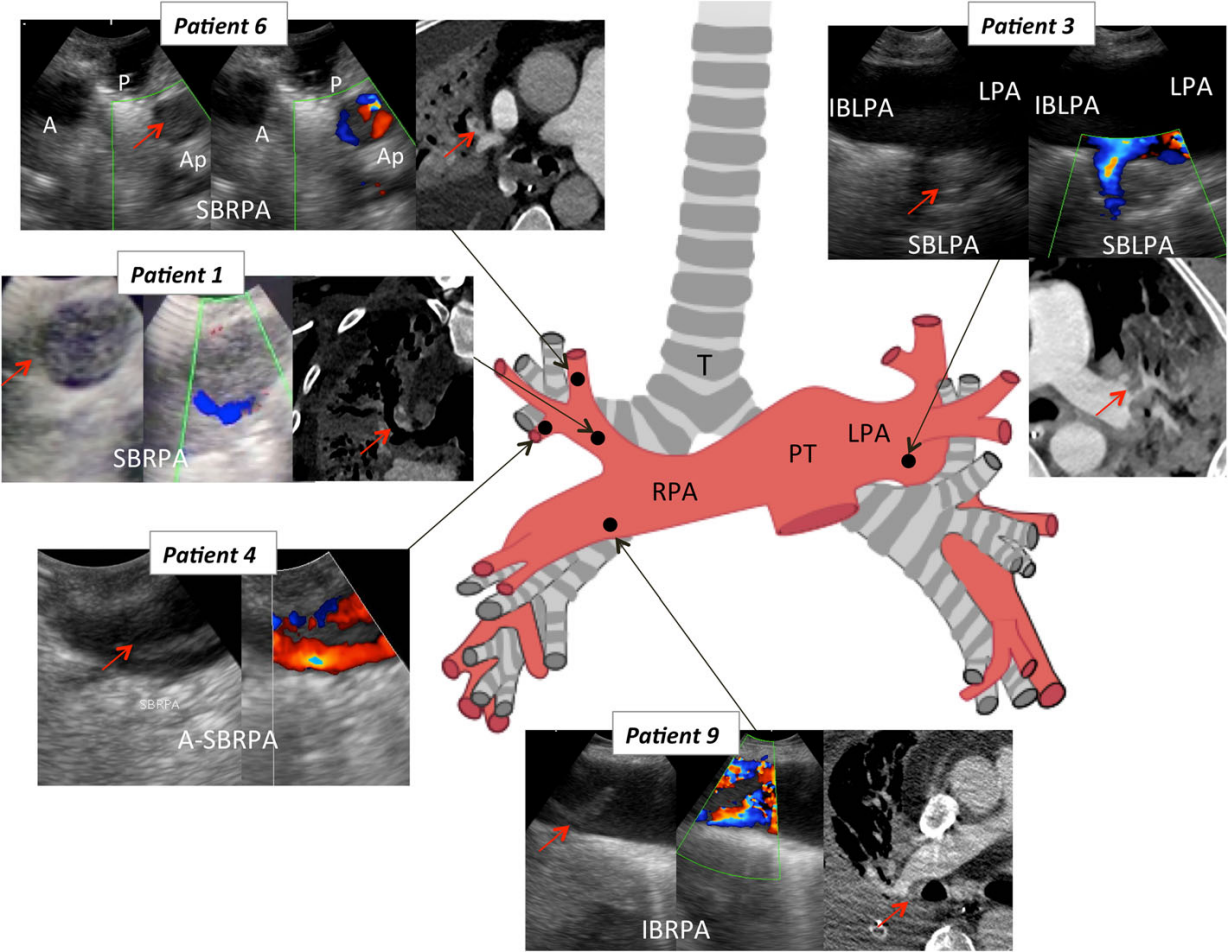
Endobronchial ultrasound and computed tomography pulmonary angiography correspondence in the five patients with pulmonary embolism. Red arrows indicate the presence of thrombus in pulmonary arteries. SBRPA, superior bronchial right pulmonary artery; A-SBRPA, anterior segment of the SBRPA; P-SBRPA, posterior segment of the SBRPA; A-SBRPA, apical segment of the SBRPA; LPA, left pulmonary artery; IBLPA, inferior bronchial left pulmonary artery; SBLPA, superior bronchial left pulmonary artery; T, trachea

Diagnostic correspondence between EBUS and CTPA is depicted in Table 1. Excluding patient 4, in which PE may have developed during the 7 days that separated EBUS and CTPA, overall agreement was obtained in 9/10 (90%) patients. The patient (*patient 2*) without PE on the EBUS had left segmental pulmonary embolism on CTPA, which was not accessible to the EBUS.

This case series of EBUS to diagnose PE in severe SARS-CoV-2 ARDS patients requiring vv-ECMO suggests that the EBUS procedure is safe and reliable to detect lobar and even segmental PE at bedside. Given the high risk of pulmonary embolism in patients with severe ARDS due to COVID-19, this minimally invasive diagnostic approach seems to be a useful and appropriate diagnostic tool to avoid the multiple adverse effects of CTPA in these severe and often unstable patients. The diagnostic performance of this innovative and promising technique needs now to be confronted to CTPA in larger prospective study and other clinical situations, especially for the analysis of segmental arteries.

## Data Availability

Our data are available to ensure transparency.

## Abbreviations

ARDS: Acute respiratory distress syndrome
Ap-SBRPA: Apical segment of the SBRPA
A-SBRPA: Anterior segment of the SBRPA
CT: Computed tomography
EBUS: Endobronchial ultrasound
IBLPA: Inferior bronchial left pulmonary artery
LPA: Left pulmonary artery
PE: Pulmonary embolism
PEEP: Positive end-expiratory pressure
P-SBRPA: Posterior segment of the SBRPA
SARS-CoV-2: Severe acute respiratory syndrome coronavirus 2
SBLPA: Superior bronchial left pulmonary artery
SBRPA: Superior bronchial right pulmonary artery
T: Trachea
vv-ECMO: Venovenous extracorporeal membrane oxygenation
